# Deletions of Chromosome 7q Affect Nuclear Organization and *HLXB9*Gene Expression in Hematological Disorders

**DOI:** 10.3390/cancers11040585

**Published:** 2019-04-25

**Authors:** Concetta Federico, Temitayo Owoka, Denise Ragusa, Valentina Sturiale, Domenica Caponnetto, Claudia Giovanna Leotta, Francesca Bruno, Helen A. Foster, Silvia Rigamonti, Giovanni Giudici, Giovanni Cazzaniga, Joanna M. Bridger, Cristina Sisu, Salvatore Saccone, Sabrina Tosi

**Affiliations:** 1 Department of Biological, Geological and Environmental Sciences, University of Catania, via Androne 81, 95124 Catania CT, Italy; federico@unict.it (C.F.); valentina.sturiale@unict.it (V.S.); domyc@live.it (D.C.); claudialeotta@live.it (C.G.L.); francescabruno@unict.it (F.B.); 2 Genome Engineering and Maintenance Network, Institute of Environment, Health and Societies, Brunel University London, Kingston Lane UB8 3PH, UK; Temi001@hotmail.co.uk (T.O.); denise.ragusa2@brunel.ac.uk (D.R.); joanna.bridger@brunel.ac.uk (J.M.B.); 3 Department of Biological and Environmental Sciences, School of Life and Medical Sciences, University of Hertfordshire, Hatfield AL10 9AB, UK; h.foster2@herts.ac.uk; 4 Associazione Italiana Ematologia Oncologia Pediatrica (AIEOP), Centro Ricerca Tettamanti, Pediatric Department, University of Milano-Bicocca, 20900 Monza, Italy; silviarigamonti91@gmail.com (S.R.); g.giudici@asst-monza.it (G.G.); gianni.cazzaniga@asst-monza.it (G.C.); 5 College of Health and Life Science, Brunel University London, Kingston Lane UB8 3PH, UK; Cristina.sisu@brunel.ac.uk

**Keywords:** genome organization, radial positioning, chromosome deletion, *HLXB9* gene, *MNX1* gene, chromosome 7, leukemia

## Abstract

The radial spatial positioning of individual gene loci within interphase nuclei has been associated with up- and downregulation of their expression. In cancer, the genome organization may become disturbed due to chromosomal abnormalities, such as translocations or deletions, resulting in the repositioning of genes and alteration of gene expression with oncogenic consequences. In this study, we analyzed the nuclear repositioning of *HLXB9* (also called *MNX1*), mapping at 7q36.3, in patients with hematological disorders carrying interstitial deletions of 7q of various extents, with a distal breakpoint in 7q36. We observed that *HLXB9* remains at the nuclear periphery, or is repositioned towards the nuclear interior, depending upon the compositional properties of the chromosomal regions involved in the rearrangement. For instance, a proximal breakpoint leading the guanine-cytosine (GC)-poor band 7q21 near 7q36 would bring *HLXB9* to the nuclear periphery, whereas breakpoints that join the GC-rich band 7q22 to 7q36 would bring *HLXB9* to the nuclear interior. This nuclear repositioning is associated with transcriptional changes, with *HLXB9* in the nuclear interior becoming upregulated. Here we report an *in cis* rearrangement, involving one single chromosome altering gene behavior. Furthermore, we propose a mechanistic model for chromatin reorganization that affects gene expression via the influences of new chromatin neighborhoods.

## 1. Introduction

The behavior of the genome within interphase nuclei gives us essential insights into the spatial and epigenetic regulation of gene expression [1,2]. The spatial positioning of different genomic regions within nuclei is non-random, and depends on the genome composition, in particular gene density and guanine-cytosine (GC)-content. The GC-richest regions, usually abundant in genes and more transcriptionally active, are positioned towards the nuclear interior, whereas the GC-poorest regions, characterized by a paucity of genes, are located towards the nuclear periphery, and are associated with gene silencing [3,4,5,6,7,8,9]. The spatial positioning of the GC-rich and GC-poor regions gives rise to a zig-zag arrangement of chromatin with a differential spatial location of individual bands that are adjacent to an individual chromosome, as demonstrated for human chromosome 7 in the cell nuclei of normal lymphocytes [5,10,11,12,13,14,15,16,17].

This nuclear distribution is further supported by evidence gathered using chromosome conformation-capture techniques such as the Hi-C method, that provide a high-resolution view of the genome in its three-dimensional (3D) organization and the interactions of higher-order chromatin structures within it [18]. These studies showed the presence of two genomic nuclear compartments, called A and B, localized at the nuclear interior (A) and at the periphery (B), that correspond to transcriptionally-active GC-rich/gene rich and transcriptionally-inactive GC-poor/gene poor compartments respectively [19,20,21]. The Hi-C methods also allowed the identification of distinct structural units called Topologically Associating Domains (TADs) with size ranging from a few kb to several Mb, defined by a higher frequency of chromatin interactions within these confined regions, with little or no interactions beyond their boundaries [22,23]. Previous studies suggested that gene silencing at the nuclear periphery is a consequence of interactions with the nuclear lamina [24,25]. In fact, certain transcriptionally-silent TADs are further organized into Lamina Associated Domains (LADs), which are genomic regions in contact with the nuclear lamina at the nuclear periphery, and are characterized by repressed or minimal transcriptional activity [26].

Although gene density/GC content seem to be the underlying factors crucial forgenome organization, in proliferating cells individual chromosomes and genes can be non-randomly relocated to other nuclear compartments during development and differentiation to accomplish specific transcriptional needs [14,27,28]. In cancer, alterations to specific chromosomes and gene locations in nuclei are particularly noticeable [29,30,31,32]. These findings imply that genomic rearrangements occurring *in cis* could also affect the 3D genome organization due to different chromosomal regions being erroneously positioned in various nuclear compartments [33,34,35,36,37].

Our earlier studies have described the localization of *HLXB9* (also known as *MNX1*), a homeobox gene mapping at 7q36.3, at the nuclear periphery in phytohaemagglutinin (PHA)-stimulated lymphocytes from healthy subjects [10], as well as its repositioning in the nucleus of leukemic cells as a consequence of a chromosomal translocation [38]. In this case, the *HLXB9* allele translocated to the chromosomal band 12p13, a region that is usually found in the inner part of the nucleus, moves toward the nuclear interior. We speculated that this repositioning of *HLXB9* is associated with its activation in leukemias with t(7;12)(q36;p13) [38,39].

*HLXB9* encodes the transcription factor HB9, involved in embryonic development for pancreatic and neuronal tissue differentiation [40,41]. While mutations of *HLXB9* are well known to be associated with the developmental disorder Currarino syndrome [42], its role in cancer is still unresolved. A number of reports have described *HLXB9* overexpression in malignancies other than leukemia, including breast cancer [43,44], prostate cancer [45], bladder cancer [46], colorectal cancer [47], liver cancer [48], neuroblastoma [28], and pancreatic tumors [49], making *HLXB9* an interesting gene in cancer biology. Owing to its function as a transcription factor, the tumorigenic activity of *HLXB9* could be attributable to the activation of erroneous transcription programs, via molecular mechanisms and subsets of target genes yet to be identified. Although the *HLXB9* gene was first discovered in B-lymphocytes, there was a lack of consensus about whether *HLXB9* is expressed in bone marrow or peripheral blood cells [50]. Still today, the expression of *HLXB9* in normal hematopoietic stem cells is debated [39,51,52,53,54].

In terms of function, *HLXB9* has been suggested to be involved in maintaining stem cell niche by regulating cell adhesion or cell-to-cell interaction genes [53], and its overexpression has been linked to induction of senescence and block in differentiation [54].

Abnormalities of chromosome 7 are frequently encountered in hematological disorders, particularly of the myeloid lineage. Deletions of the long arm of chromosome 7, del(7q), are commonly found in the myelodysplastic syndrome (MDS), regarded as a form of pre-leukemia, and acute myeloid leukemia (AML) [55]. These deletions vary in extent from patient to patient, and can encompass several chromosomal bands [56,57,58,59]. Monosomy 7 and del(7q) are also found in Fanconi anemia (FA) patients who progress to develop MDS or AML [60]. Moreover, the majority of deletions are interstitial, meaning that the 7q telomere and subtelomeric region, including the *HLXB9* gene, are retained.

Understanding the chromosome 7 biology in leukemia is of interest both from a clinical and mechanistic perspective. A number of candidate genes possibly involved in leukemogenesis are found in the 7q region [55], with *HLXB9* arousing particular interest. These observations prompted us to investigate the radial nuclear positioning of *HLXB9* in the nuclei of cells from hematological patients harboring deletions of different sizes and breakpoints along the long arm of chromosome 7. In this study, we investigated the behavior of the *HLXB9* gene in hematological disorders, including expression patterns in a large patient cohort. In particular, we aim to address a number of key questions surrounding the altered nuclear positioning of *HLXB9* in leukemia with deletions of chromosome 7, and to identify any associations between the observed location, and changes in gene expression, size of the deletion, and cytogenetic mapping of affected chromosomal bands.

## 2. Results

### 2.1. Data Mining of HLXB9 Expression

**Figure 1:**
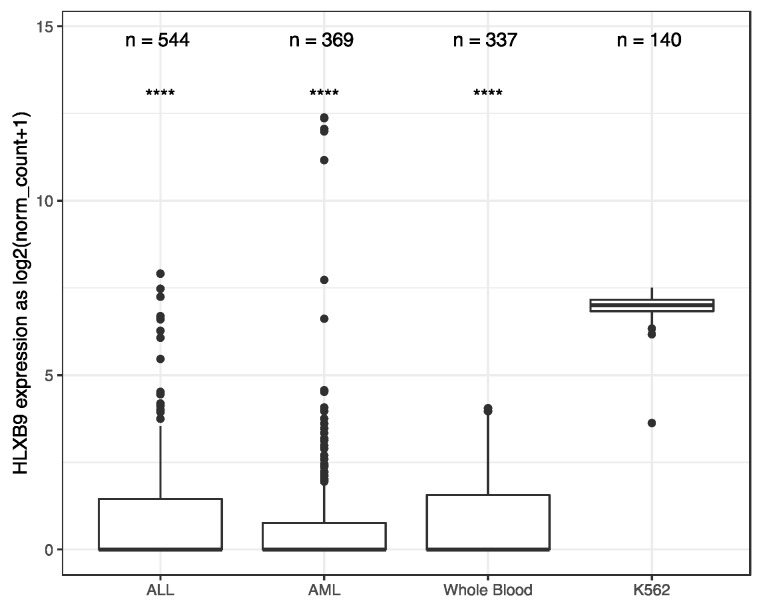
*HLXB9* expression-level distribution in acute myeloid leukemia (AML), acute lymphoblastic leukemia (ALL), whole blood and K562 leukemia cell line. The number of samples in each dataset is given above. Whole blood and K562 samples were extracted from Genotype-Tissue Expression (GTEx). AML is a composite dataset combining cross-project normalized samples from The Cancer Genome Atlas (TCGA)(173 samples) and the Therapeutically Applicable Research to Generate Effective Treatments (TARGET) (196 samples). **** *p* < 0.0001.

**Figure 2:**
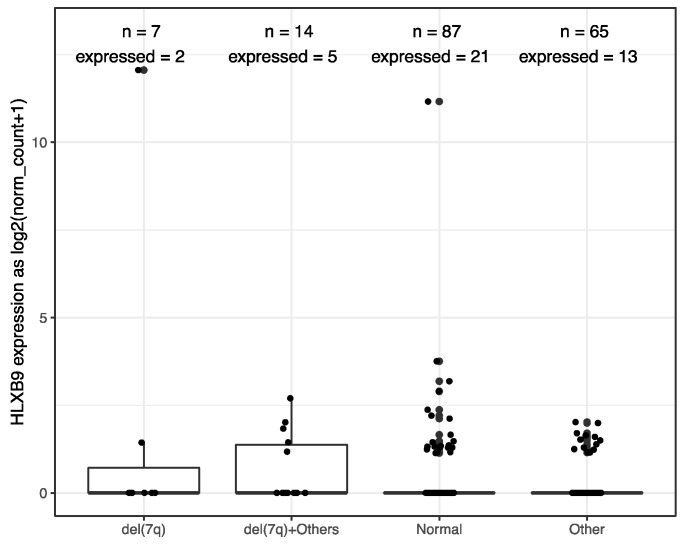
*HLXB9* expression level distribution in 173 TCGA AML samples stratified by cytogenetic phenotypes, where “n” signifies the total number of samples in each class, while “expressed” indicates the number of samples with *HLXB9* expression level higher than 1.

We investigated the expression of *HLXB9* in acute myeloid leukemia (AML) and Acute lymphoblastic leukemia (ALL) by taking advantage of the available data from The Cancer Genome Atlas (TCGA), and the Therapeutically Applicable Research to Generate Effective Treatments (TARGET) initiative. We extracted and analyzed 369 AML and 544 ALL samples, of which 173 AML samples form TCGA had associated information regarding their cytogenetics. Data from 337 whole blood samples and 140 replicates of the K562 leukemia cell line from the Genotype-Tissue Expression (GTEx) project were used as negative and positive controls, respectively, for *HLXB9* expression. Overall, similar levels of *HLXB9* expression are observed in the majority of samples in ALL, AML and whole blood, when compared to the K562 cell line, which shows significantly higher expression (Figure 1, Supplementary Materials Figures S1,S2). However, a number of samples within the ALL and AML cohorts were distinguishable by a higher expression of *HLXB9* compared to whole blood. We explored whether this may be associated with the cytogenetic features in the AML samples (cytogenetic data was unavailable for ALL). Using a cut-off value of 1 for the log2 normalized expression level, we were able to identify 7 samples characterized by the presence of del(7q) with high *HLXB9* expression. Detailed phenotypic information of these cases is reported in Table S1. We subdivided the entire AML cohort in 4 classes based on the presence of a del(7q), according to the reported cytogenetic abnormality (Figure 2). Overall, about 30% of samples with a del(7q), alone or in conjunction with other abnormalities, shows expression of *HLXB9*. A detailed overview of *HLXB9* expression levels across all cytogenetic groups is shown in Supplementary Materials Figure S3.

### 2.2. Classification of Large Series of Patients Based on HLXB9Expression Patterns

**Figure 3:**
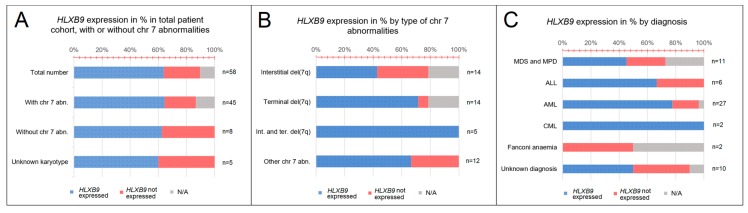
*HLXB9* expression patterns in the patients analyzed. (**A**) Graph showing proportion of patients with *HLXB9* expression categorised by the type of hematological disorder determined at the time of diagnosis. (**B**) Graph showing *HLXB9* expression in the total number of patients considered in this study, categorised into three groups: Patients with or without chromosome 7 abnormalities, and patients with an unknown karyotype. (**C**) *HLXB9* expression in patients with chromosome 7q abnormalities by subtype (interstitial, terminal, interstitial and terminal, and other).

Altogether, we collected a total of 58 samples from patients with various hematological disorders, including myelodysplastic syndrome (MDS) and myeloproliferative disorder (MPD) (*n* = 11), acute lymphoblastic leukemia (ALL) (*n* = 6), acute myeloid leukemia (AML) (*n* = 27), chronic myeloid leukemia (CML) (*n* = 2), and Fanconi anemia (FA) (*n* = 2) (Figure 3A). Expression of *HLXB9*, assessed by reverse transcription polymerase chain reaction (RT-PCR), was detected in 15 samples out of 58. For 6 samples the expression of *HLXB9* is unknown, due to lack of sufficient material. According to the karyotypes available, we subdivided the entire cohort into patients with chromosome 7 abnormalities (*n* = 45), and patients without chromosome 7 abnormalities (*n* = 8); for 5 patients the karyotype was unavailable. 10 patients with chromosome 7 abnormalities expressed *HLXB9*(29 did not express *HLXB9*, 6 N/A); 3 patients without chromosome 7 abnormalities out of 8 were positive (Figure 3B).

We further subdivided the chromosome 7 abnormality cohort by type—patients with interstitial deletions (*n* = 14), terminal deletions (*n* = 14), interstitial and terminal deletions (*n* = 5), and other (e.g., insertions, additions, ring, etc.) (*n* = 12). When these groups were investigated for the expression of *HLXB9*, 5 patients had an interstitial deletion of 7q; 1 had a terminal deletion, and 4 had other types of chromosome abnormality (Figure 3C). By classifying the patients based on their diagnosis, we found that *HLXB9* was expressed in 3 patients diagnosed with MDS or MPD, 2 with ALL, 5 with AML, and 1 with FA. Out of 10 patients with an unknown diagnosis, 4 cases showed *HLXB9* expression (Figure 3A).

### 2.3. Deletion Mapping of Breakpoints, Radial Nuclear Location and Expression Analyzes in a Selected Series of Patients

From the series of 58 patient samples, we selected 10 (referred to as Pt-1 to Pt-10) with interstitial deletions of the long arm of chromosome 7, for which we had good quality material to enable us to conduct further molecular cytogenetic characterization and expression analysis. The clinical and cytogenetic details are reported in Table 1.

#### 2.3.1. Characterization of del(7q) Breakpoints

**Figure 4:**
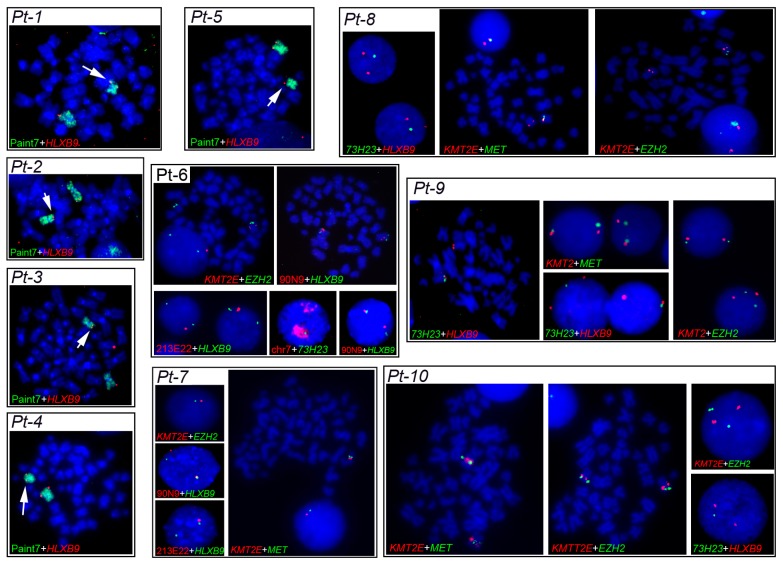
Fluorescence in situ hybridization (FISH) patterns observed in metaphases and nuclei of patients with del(7q). The 7q deletion breakpoints in Pt-1 to Pt-10 were determined by dual-color FISH using different probes, indicated at the bottom of each panel. The *HLXB9* gene was detected using the BAC probe RP5-1121A15 (7q36.3). KMT2E (7q22) + MET (7q31.2) and KMT2E + EZH2 (7q36.1) were detected using commercial probe mixtures from MetaSystems. Whole chromosome paint 7, RP11-90N9 (7q21.11), RP11-211E22 (7q22.1), and RP11-73H23 (7q34) are BAC probes previously described (see Materials and methods). Metaphase chromosomes and interphase nuclei were counter-stained in blue with DAPI. Magnification 1000×.

**Figure 5:**
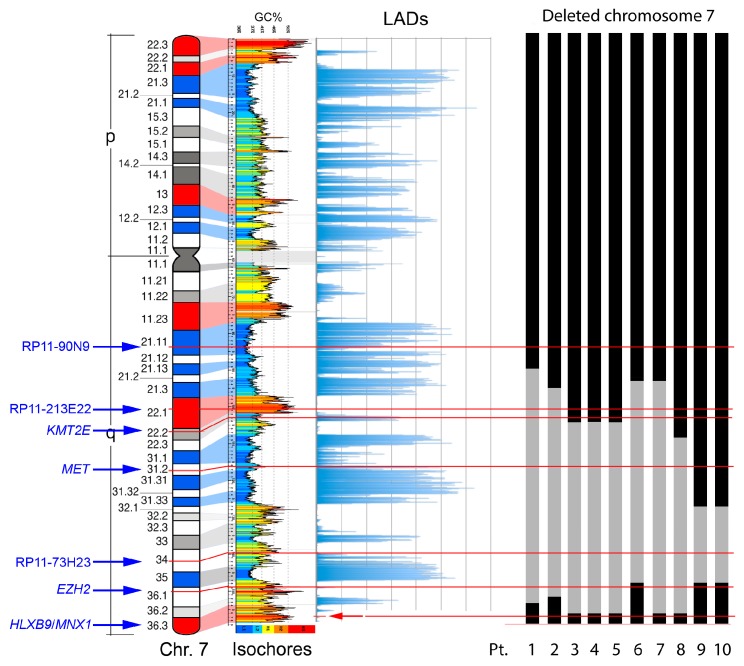
Genomic properties of chromosome 7 and the extent of the deleted region in the del(7q) patients. From left to right: Probes for the main landmarks used for FISH experiments, ideogram of the chromosome 7 showing the guanine-cytosine (GC)-richest (red) and the GC-poorest (blue) bands [10],correspondence with the GC-level of the genomic DNA sequence [62], distribution of the Lamina Associated Domains (LADs) along the chromosome [63], and schematic representation of the deleted region (in grey) in the del(7) chromosome detected in the ten subjects analyzed (Pt.: Patient number and deletion data from Table 1 and Table 2). The thin horizontal lines indicate the position of the probes indicated on the left.

Characterization of deletion breakpoints relied on both conventional karyotype analysis at the time of diagnosis (Table 1), and FISH (Table 2). We previously used FISH to map precisely the breakpoints in Pt-1 to Pt-4 in our earlier studies [58,61]. Here, for Pt-5 to Pt-10, we used FISH with probes localized along the long arm of chromosome 7 to determine the proximal and distal breakpoints of the del(7q), and whether the *HLXB9* locus is retained in the deleted chromosome, to refine the karyotypes in some cases. For Pt-4 and Pt-5, the samples were of insufficient amount to allow further experiments needed to establish more precise breakpoints. Representative FISH images are shown in Figure 4. The presence of two hybridization signals for the locus-specific probe for *HLXB9* (RP5-1121A15) at 7q36.3 in all 10 patients confirmed that *HLXB9* was retained in all cases. In Pt-1 to Pt-5, this is shown in metaphases by the presence of a deleted chromosome 7 shorter than the normal homolog painted in green, and both *HLXB9* loci detected in red by RP5-1121A15. In Pt-6 to Pt-10, two *HLXB9* alleles are shown in metaphases, and nuclei hybridized by the same probe. Hybridization with the probe mixture containing the EZH2 locus at 7q36.1 further narrowed down the distal breakpoint in Pt-7 and Pt-8, which was mapped at 7q36.2, due to the absence of signals for this probe on the deleted chromosome. Pt-6, Pt-9 and Pt-10 had both signals for EZH2, indicating that the distal breakpoint lies on a region proximal to it, but could not be further determined. In Pt-6 to Pt-10, the 7q21.11 region was retained, as shown by two signals for the RP11-90N9 probe. Pt-6 and Pt-7 showed loss of the 7q22 and 7q31 regions on the deleted chromosomes, as shown by single signals for probes containing KMT2E (7q22), RP11-213E22 (7q22.1) and MET (7q31.2), indicating that the breakpoint lies in 7q21. These loci, however, were retained in Pt-9 and Pt-10. The breakpoint in Pt-8 was mapped at 7q22, considering that the 7q34, and 7q31.2signals were lost. In Pt-9 and Pt-10, signals for the 7q31 region were present, placing the proximal breakpoint further away, compared to other patients, approximately between 7q32 or 7q33. Given that only one signal for RP11-73H23 at 7q34 is seen in Pt-6 to Pt-10, and that the *HLXB9* locus at 7q36.3 is consistently kept, we concluded that the deletions were all interstitial, with the distal breakpoint in 7q35/q36, and a variable proximal breakpoint between 7q21 and 7q33. Together with our previous analysis of Pt-1 to Pt-4 [58,61], we can summarize the proximal breakpoints as follows: In the 7q21 band in Pt-1, Pt-2, Pt-6 and Pt-7; in the 7q22 band in Pt-3, Pt-4, Pt-5 and Pt-8; in the 7q32/33 region in Pt-9 and Pt -10. A graphical view of the breakpoints in each patient, and the GC-content along chromosome 7 is shown in Figure 5.

#### 2.3.2. *HLXB9* Radial Nuclear Location

**Figure 6:**
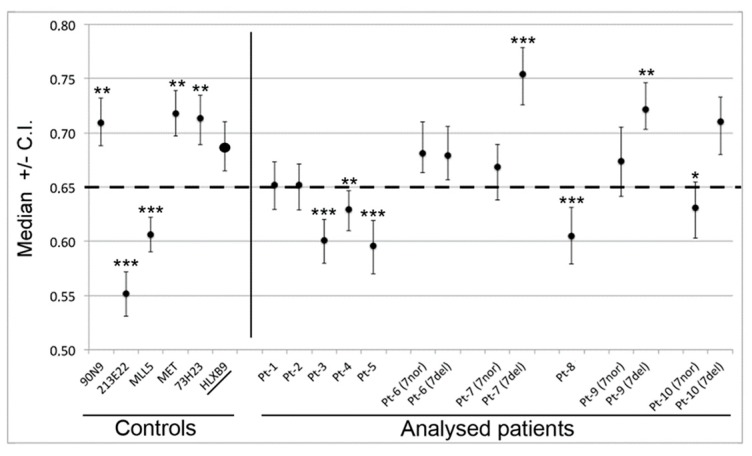
Radial nuclear location (RNL) of the *HLXB9* locus in del(7q) patients. The graph displays median value and relative confidence interval (C.I.) corresponding to the RNL of the *HLXB9* locus in patients and controls. On the left are the RNL of the genomic loci 90N9 (7q21.11), 213E22 (7q22.1), KMT2E (7q22.3), MET (7q31.2), 73H23 (7q34) and *HLXB9* in nuclei of control cells (lymphocytes from peripheral blood of volunteer healthy donors from [10]). On the right side, each RNL value refers to the *HLXB9* gene in the indicated patients (Pt-1 to Pt-10). For Pt-6, Pt-7, Pt-9, and Pt-10 the two alleles were measured separately: 7nor and 7del indicate *HLXB9* in the normal or deleted chromosome 7, respectively. In the other cases, the RNL value refers to both *HLXB9* alleles, as it was not possible to discriminate between the two, due to a low number of informative nuclei. The dashed line at 0.65 indicates the median value separating the peripheral and the internal nuclear compartment, i.e. values higher than 0.65 are peripheral, and values lower than 0.65 are internal to the nucleus. Statistical significance of the difference in the RNL data obtained for each probe with respect to the *HLXB9* in the control cells (name underlined, and RNL indicated by an enlarged spot), was evaluated using the two-tailed *t* tests. *p* values are: * *p* < 0.05, ** *p* < 0.001, *** *p* < 0.0001.

We determined the radial nuclear location (RNL; expressed as median values) of the 7q36 region encompassed by RP11-1121A15 (containing the *HLXB9* gene) in the 10 selected patient samples (Figure 6). Observations were carried out on both homologs, normal chromosome 7 and deleted chromosome whenever possible, depending on sample quality. Data for Pt-1 to Pt-5 were obtained using single-color FISH with the probe RP11-1121A15. This approach did not enable us to discriminate between the normal chromosome 7 and the deleted one. As a consequence, data are skewed by the presence of the *HLXB9* allele on the non-deleted chromosome. The use of dual-color FISH in the other cases (Pt-6 to Pt-10) enabled us to discriminate between the two chromosomes, hence to obtain the individual RNL of the two *HLXB9* alleles (“7nor” and “7del” in Figure 6). For Pt-8, although a dual-color approach was used, the RNL was calculated based on both alleles, since it was not possible to clearly identify the allele on the deleted chromosome from the non-deleted one. Overall, our analysis revealed that for Pt-1, Pt-2, Pt-6, Pt-7, Pt-9 and Pt-10, *HLXB9* is located in the peripheral region of the nucleus (i.e. RNL higher than 0.650), which is the same compartment where the gene is located in the controls phytohaemagglutinin (PHA)-stimulated lymphocytes (left panel in Figure 6). By contrast, Pt-3, Pt-4, Pt-5 and Pt-8 showed that *HLXB9* was positioned more internally in the nucleus (i.e. RNL lower than 0.650), compared to the controls (statistical differences highly significant, *p* < 0.0001 for Pt-3, Pt-5 and Pt-8 and *p* < 0.001 for Pt-4) (Figure 6). In the cases where we were able to distinguish between the normal and deleted chromosomes (i.e. Pt-6, Pt-7, Pt-9 and Pt-10), *HLXB9* in the deleted chromosome 7 of Pt-7 (RNL = 0.754) and Pt-9 (RNL = 0.722), is located even more peripherally compared to the controls, and compared to the allele belonging to the non-deleted chromosome (*p* < 0.0001 and *p* < 0.001, respectively). *HLXB9* in Pt-6 maintains a nuclear location similar to that of the control, and there is no significant difference between the normal and deleted chromosomes. In Pt-10 the allele located in the normal chromosome 7 is more internal than the control (*p* < 0.05).

#### 2.3.3. *HLXB9*Expression

**Figure 7:**
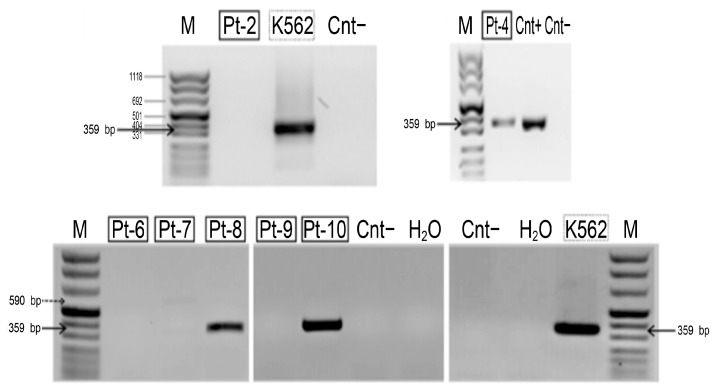
Detection of the *HLXB9* transcript in patient samples with del(7q). Agarose gel electrophoresis containing nested reverse transcription (RT)-PCR products obtained using primers specific for *HLXB9*. The molecular marker (M) is peqGOLD DNA-Sizer XI (range 67-1118 bp, PeqLab, Fareham, UK). The amplified DNA fragment, indicated by black arrows, corresponds to theexpected size of the *HLXB9* transcript (359 bp). The transcript is clearly present in Pt-4, Pt-8 and Pt-10. Positive controls are the leukemia-derived cell line K562 and another patient sample (Cnt+), known to express *HLXB9* (patient no. 10 in [38]).

*HLXB9* expression analysis revealed the presence of the *HLXB9* transcript in Pt-4, Pt-8, and Pt-10, but not in Pt-2, Pt-6, Pt-7, and Pt-9 (Figure 7). Insufficient material from Pt-1, Pt-3 and Pt-5 did not enable us to carry out the analysis in these samples. The amplified transcript corresponds to the predicted fragment of 359 bp, demonstrating the presence of the *HLXB9* transcript in these samples. By comparing HLXB9 expression with its radial nuclear location and the GC-content of the chromosomal region in the vicinity of the proximal brakpoint, we observed a high degree of concordance of these three parameters: (1) HLXB9 is expressed, (2) HLXB9 is localized in the nuclear interior, and (3) high GC-level of the involved chromosomal region; or, in the opposite case, (1) HLXB9 is not expressed, (2) HLXB9 localizes at the nuclear periphery, and (3) low GC-level of the involved chromosomal region (Table 3).

## 3. Discussion

Increased expression of the homeobox gene *HLXB9* has been described in several cancers, including leukemia [28,38,39,43,44,45,46,47,48,49,52]. However, little is known about the oncogenic mechanisms of *HLXB9*. In a specific type of infant leukemia, *HLXB9* transcription seems to be triggered by the chromosomal rearrangement t(7;12)(q36;p13), where the breakpoint on chromosome 7 lies proximal to *HLXB9* [64]. It has been proposed that *HLXB9* may act as a tumor suppressor in AML, and oncogene in ALL [65]. Our interests focus on understanding gene repositioning in the context of nuclear architecture and genome organization as possible mechanisms of *HLXB9* activation in hematological malignancies. In this study, we aimed to address (i) whether there are consistent patterns of *HLXB9* expression in a broad variety of hematological disorders, (ii) if *HLXB9* expression is associated with the presence of chromosome 7 abnormalities, (iii) if *HLXB9* expression in del(7q) patients varies depending on chromosomal breakpoints, (iv) if *HLXB9* expression status is associated with the positioning of the gene in particular nuclear compartments.

### 3.1. HLXB9 Expression is Not Associated with Any Specific Leukemia Subtype

In this study, we looked at *HLXB9* expression in publicly-available databases (TCGA, TARGET and GTEx; Figure 1 and Figure 2), and in a cohort of 58 patients with various hematological disorders by RT-PCR (summarized in Figure 3). Across the datasets, *HLXB9* expression in the majority of samples was comparable to that of normal blood. However, it was evident that a number of outliers are spread at higher expression levels in AML and ALL, suggesting that *HLXB9* expression could be linked to features other than diagnosis. Similarly, data gathered from our 58 patient samples showed *HLXB9* expression in approximately 20% of cases. From our initial survey of the total patient cohort according to diagnosis (Figure 3A), the patterns of *HLXB9* expression varied, and was not particularly prominent in any diagnostic group. A higher number of positive patients was noticed in the MDS-MPD and AML groups, consistent with the higher incidence of chromosome 7 abnormalities in these disorders. The small sample sizes for the two patients with FA limited our interpretation for *HLXB9* expression in this subgroup. These observations are in line with the description of *HLXB9* overexpression in a variety of malignancies unrelated to the hematopoietic system, some of which correlate with the role of *HLXB9* in pancreatic and neuronal development in the embryo [28,43,44,45,46,47,48,49].

### 3.2. HLXB9 Expression is Not Necessarily Associated with the Presence of Chromosome 7 Abnormalities

The expression of *HLXB9* according to cytogenetic subgroups in public databases correlated with our observations in the patient cohort (Figure 2 and Figure 3). Approximately 30% of patients with del(7q), alone or in conjunction with other rearrangements, showed expression of *HLXB9*, when compared to 20–25% of normal karyotypes or other subgroups. The expression of *HLXB9* in patients without chromosome 7 abnormalities indicates that its activation may not be solely dependent on chromosome 7 rearrangements. It should be taken into consideration that a proportion of patients presents cryptic abnormalities or microdeletions of 7q [39,66], which may go unnoticed by conventional karyotyping, and become misclassified in the studied cohorts. Similarly, it cannot be excluded that rearrangements of other chromosomes may bring about changes in nuclear organization that indirectly activate other genes *in trans*. However, among patients with chromosome 7 abnormalities in our cohort, *HLXB9* was expressed at a higher proportion in patients with interstitial deletions of 7q, and those with other chromosome 7 abnormalities (e.g. rings, additions, translocations), when compared to patients with terminal deletions (i.e. with loss of the *HLXB9* allele on the deleted chromosome). It was not possible to compare this data with published datasets due to the lack of cytogenetic information in the latter.

### 3.3. HLXB9 is Expressed When del(7q) Proximal Breakpoint Lies in a GC-rich Genomic Region

The 10 samples used in the present work were characterized by an interstitial deletion of the long arm in one of the two chromosomes 7. The deletions spanned from few to several chromosomal bands, and the deleted segment did not include the *HLXB9* gene, located at 7q36.3, at about 2.3 Mb from the telomere (Figure 5). Each deletion determined a specific repositioning of *HLXB9* in the nucleus, depending on the GC-content of the chromosomal region close to the proximal breakpoint. We compared the *HLXB9*expression status of each patient with the RNL of *HLXB9* and the compositional properties of the chromosomal region close to the centromeric breakpoint, observing a large degree of concordance with these three parameters (Table 3). We observed that deletions causing a repositioning of the 7q36 region near the GC-poor bands 7q21 (Pt-1, Pt-2, Pt-6 and Pt-7) or 7q31 (Pt-9 and Pt-10), result in a more peripheralnuclear localization of the *HLXB9* allele. Conversely, deletions joining the telomeric end of chromosome 7 with the GC-rich 7q22 band (Pt-3, Pt-4, Pt-5 and Pt-8) result in an inner nuclear localization of *HLXB9*.

Thus, the transcriptional activation of *HLXB9* occurs only in those cases carrying a deletion of chromosome 7 where the gene moves to the more internal part of the nucleus. Indeed, two cases (Pt-4 and Pt-8) showed a relocation of *HLXB9* in the inner part of the nucleus and activation of its transcription. Incidentally, in Pt-10, *HLXB9* expression was also observed, but the allele re-located to the inner part of the nucleus belongs to the non-deleted chromosome 7. In this latter case, we can hypothesize that transcriptional activation could be due to anectopic activation of this allele, and may indicate a more complex mechanism leading to gene activation in leukemia. We described a similar scenario in the leukemia cell line GDM-1 where, in the presence of the translocation t(6;7)(q23;q36), the activation of *HLXB9* seems to arise from the non-affected chromosome [32]. It is to be noted that it is still a topic of debate whether *HLXB9* is active in non-pathological conditions and in certain differentiated cells, other than during embryonic development [39,51,52,53,54].

### 3.4. Mechanisms Leading to HLXB9 Re-positioning in the Nucleus

**Figure 8:**
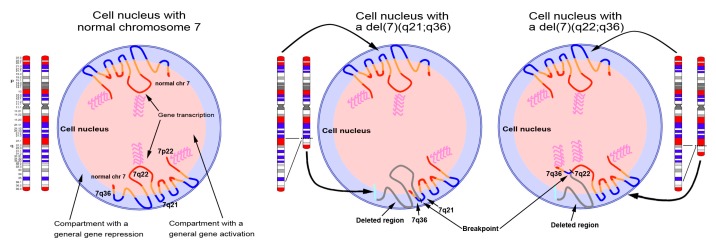
Distribution of chromosome 7 genomic material across the nucleus. The illustration depicts three nuclei with two normal copies of chromosome 7 (**left**), one chromosome 7 with an interstitial deletion involving the region included between 7q21 and 7q36 (middle), and one chromosome 7 with an interstitial deletion involving the region included between 7q22 and 7q36 (**right**). The representation of the chromosome 7 territories follows a zig-zag conformation of the DNA, according to data previously published [10]. Depending on the extent of the deletion, the terminal region of 7q, harboring *HLXB9*, becomes juxtaposed to either the 7q21 band or the 7q22 bands. The vicinity to the 7q21 band, positioned at the periphery of the nucleus, determines a peripheral position for *HLXB9*, whereas the vicinity to the 7q22 band, normally positioned towards the inner part of the nucleus, causes an alteration in the location of *HLXB9*, which becomes positioned more internally. Since the repositioning potentially alters the gene expression pattern of the affected gene, this could explain the ectopic activation of *HLXB9* allele repositioned in the inner part of the nucleus. Red and blue chromosomal bands correspond to the GC-richest and the GC-poorest bands in the chromosome 7 [10].

Although the biology of hematological disorders with del(7q) is still largely unknown, the del(7q) seems to have an important role in tumor emergence and progression [67,68,69]. We speculate that the oncogenic mechanisms of del(7q) leukemia rely on differences in gene expression patterns that reflect different deletion breakpoints (Figure 8). Deletions involving different chromosomal bands might lead to the activation or silencing of different gene pathways due to alteration of the nuclear architecture and genome organization.

According to our results, the repositioning of *HLXB9* in the nucleus of leukemia cells is dependent on the newly juxtaposed chromosomal region resulting from the deletion. Our observations are consistent with other reports showing that the repositioning of genes is non-random, and depends on the properties of neighboring genomic loci [70]. 

Little is known regarding the mechanisms that induce gene repositioning in the nucleus. Zones of gene deserts seem to be enriched with lamina-associated domains (LADs) that favor their attachment to the nuclear lamina, resulting in their transcriptional silencing [26,71]. The distribution of such LADs along different regions of chromosome 7 (Figure 5) [63] further supports the model ofzig-zag positioning of chromosome 7 in the nuclei of control lymphocytes (Figure 6) [10]. Theoretically, an interstitial deletion bringing together two zones of transcriptionally silent LADs at the nuclear periphery would not produce an effect on the expression of a particular gene. Similarly, a loss of LADs could prevent an adjacent region from interacting with the nuclear lamina, and becoming transcriptionally active under a new neighboring structural domain. 

### 3.5. Gene Expression in 3D Chromatin Architecture

Individual gene repositioning within the interphase nucleus should be seen in context with the more complex 3D organization of the whole genome and chromatin architecture in differentiated or replicative cells. It was recently shown that evolutionarily successful chromosomal rearrangements do not alter the nuclear position of the regions involved. This is not the case in pathological situations, where the rearrangements typically alter the gene structure, and often also the gene nuclear position [32].

Many studies have focused on how gene relocation might affect gene regulation and expression, in both cell development and disease [14,16,28,38,72,73], and how these alterations are associated with the interaction with certain structures such as LADs and TADs [24,74,75]. We foresee that these interactions, higher-order chromatin features, and their disturbance caused by chromosomal rearrangements, are the key to understanding the oncogenic changes in gene expression, which would bring us a step closer in comprehending the process of leukemogenesis associated with chromosome 7 aberrations.

Current research on spatial organization of the genome in cancer cells confirms that a number of genes are consistently repositioned in specific pathologies, such as breast and prostate cancer [30,31,76,77,78]. Therefore, the analysis of nuclear gene positioning in cancer could be of diagnostic value. Some investigations also showed repositioning of whole chromosomes in leukemia samples prior to relapse, indicating the prognostic value of nuclear positioning studies [79]. Further work is needed to fully understand the implications of our findings, and if mapping of *HLXB9* in the nucleus of leukemia cells has any diagnostic potential. The prognosis of del(7q) leukemias is known to be generally very poor, irrespective of breakpoint or extent of deletion. Similarly, the presence of del(7q) in MDS-MPD and FA patients is a sign of malignant transformation, and hence indicative of poor clinical outcome. However, the added information coming from *HLXB9* repositioning, associated with gene expression data, might help to refine the classification of these cases, and aid design tailored therapy.

## 4. Materials and Methods 

### 4.1. Patient Samples

We used archival material from 58 patient samples in the form of fixed chromosome and nuclei suspensions in methanol:acetic acid, some of which were used in previous studies [38,58,61]. Of these, we selected 10 samples (Pt-1 to Pt-10, with details in Table 1) with good material quality, enabling us to carry out more detailed analyzes using fluorescence in situ hybridization (FISH) with several genomic probes and reverse transcription polymerase chain reaction (RT-PCR), to assess *HLXB9* expression. Four patient samples presented in this work (from Pt-1 to Pt-4) were described in earlier reports [38,58,61]. Archival materials of the six other patients (from Pt-5 to Pt-10) are described here for the first time. Pt-5 was contributed by Professor Jochen Harbott, Oncogenetic Laboratory, Children’s Hospital, University of Giessen, Germany. Pt-6 to Pt-10 were contributed by Associazione Italiana Ematologia Oncologia Pediatrica (AIEOP), Centro Ricerca Tettamanti, Pediatric Department, University of Milano-Bicocca, Monza, Italy. A complete karyotype, available in the majority of cases, was obtained using chromosome banding with standard methods. Moreover, the extent of deletions was refined by FISH. Ethical approval no: 16516-TISS-Apr/2019-18741-2.

### 4.2. Control Samples

Control samples from peripheral blood lymphocytes of healthy individuals were prepared and analyzed previously [10]. Data of radial nuclear location (RNL) published at that time was used as a reference for the present study. Furthermore, the myeloid leukemia cell line K562, and a patient positive for *HLXB9* expression (patient no. 10 described in previous work [38]), were used as positive controls for expression analysis based on RT-PCR performed on Pt-6 to Pt-10.

### 4.3. Reverse Transcription Polymerase Chain Reaction (RT-PCR)

RNA was extracted from archival fixed-chromosome and nuclei suspensions using Qiagen RNeasy mini Kit (Qiagen, Manchester, UK). cDNA was synthesised using Superscript III reverse transcriptase kit (Life Technologies, Paisley, UK). Nested RT-PCR was conducted to assess *HLXB9* expression using primers and conditions as previously described [38]. Briefly, primers for the first round of amplification were HB9-WT1f: 5’-CTTCCAGCTGGACCAGTGGCTG-3’ and HB9-WT1r: 5’-CGTCCTCGTCCTCGTCCTCC-3’, and HB9-1994 forward: 5’-TCCACCGCGGGCATGATCCTG-3’ and HB9-WT2 reverse: 5’-GGCCCCAGCAGCTCCTCGGCTC-3’ for the second round. 

The amplification conditions were: Denaturation at 94°C for 3 min, followed by 35 cycles of annealing at 68 °C for 30 s, extension at 68 °C for 1 min, final extension at 68 °C for 3 min, holding at 4°C. The predicted size of the amplified DNA segment is 359 bp, based on the available sequence of the *HLXB9* mRNA (accession number: NM_005515.4), also consistent with the results shown by [80].

### 4.4. Data Mining

Data from the three datasets. The Cancer Genome Atlas (TCGA), Therapeutically Applicable Research to Generate Effective Treatments (TARGET) and Genotype-Tissue Expression (GTEx), were obtained from the University of California, Santa Cruz, Xena repository. The raw gene expression data across the three datasets (TCGA, TARGET, and GTEx) was processed at UCSC using the TOIL pipeline as described in [81]. All the expression levels for the genes of interest were extracted from the TCGA-TARGET-GTEX cohort. To investigate the association between expression level and the cytogenetic phenotype, we extracted phenotypic information from the TCGA acute myeloid leukemia (AML) and Therapeutically Applicable Research to Generate Effective Treatments (TARGET) acute lymphoblastic leukemia (ALL)datasets. 7q deletion information was available only for the TCGA AML data. We used 337 whole blood and 140 K562 Leukemia cell line sample data from the Genotype-Tissue Expression (GTEx) project as controls.

### 4.5. Fluorescence in Situ Hybridization (FISH) and Image Capture

Metaphase chromosome and interphase nuclei from the patients analyzed here were obtained from the archival material stored in methanol-acetic acid. Dual-color FISH experiments on metaphase chromosomes and interphase nuclei were performed using a selection of probes mapping at different sites in the long arm of chromosome 7 (see Table 2 for list of probes and corresponding chromosomal location). In particular, for the mapping of *HLXB9* on 7q36.3, we used the PAC RP5-1121A15 (GenBank accession No. AC006357.5) containing the gene. We also used commercially available probe mixtures XL 7q22/7q36, containing the KMT2E and EZH2 genes, and XL del(7)(q22q31), containing the KMT2E and MET genes (MetaSystems, Athlussein, Germany). Other probes included chromosome 7 paint directly labeled in green (Cambio, Cambridge, UK), BACs RP11-90N9 (GenBank accession No. AZ518618.1), RP11-213E22 (GenBank accession No. AQ484445.1), RP11-73H23 (GenBank accession No. AQ266610.1). Additionally, we included probes previously used and described in [58]. These were: Cosmid clones Cos5.3, Cos7d.2, Cos8a.2, Cos1107, Cos1120 and YAC clones HSC7E485, HSC7E124, HSC7E86, HSC7E769, and the PAC clone 1065. Probes were labeled either directly with fluorochromes, or indirectly with biotin or digoxigenin, using nick translation (Roche, Mannheim, Germany), and detected according to standard methods previously described [21]. FISH experiments were carried out as previously described [10,82]. Hybridization signals on metaphase chromosomes and interphase nuclei were analyzed using an Olympus AX70 fluorescence microscope, and images were captured using MacProbe v4.3 software (Applied Imaging, Newcastle, UK).

### 4.6. Radial Nuclear Positioning Analysis

Radial nuclear location (RNL) of *HLXB9* and other relevant loci was based on two dimensional (2D) analysis of FISH images of interphase nuclei as previously described [10,21]. Briefly, the RNL of each hybridization signal was determined as the ratio of the nuclear radius. The numerical value (namely the position of the hybridization signals along the nuclear radius) ranges between 0 and 1, where 0 indicates the cent and 1 the outer extreme of the nucleus. Depending on sample quality, we aimed at scoring at least 200 nuclei per sample. The localization of large number of hybridization signals relative to specific loci was determined according to well-established statistical methods that take into consideration the median value of all signals +/− confidence interval (CI) [10]. According to these methods, median values lower than 0.650 are indicative of loci positioned towards the nuclear interior [10,28,32,38]. 

The statistical analysis and the corresponding graphs were carried out using Microsoft Excel and StatView software (SAS Institute Inc., Cary, USA) [83]. Statistical significance was evaluated using the Two-tailed *t*-test.

## 5. Conclusions

In this study, we address the radial nuclear repositioning of a gene in relation to the compositional properties of chromosomal bands involved in a chromosomal deletion and its effect onits transcriptional activation. We show here that the repositioning of *HLXB9* in the nuclei of leukemia cells with del(7q) depends on the location of the breakpoints on chromosome 7, which implies the disruption and re-joining of different chromosomal bands. This type of repositioning may influence the nuclear organization of the chromatin and the transcriptional activity of the repositioned genes. We show that *HLXB9* becomes activated when relocated to the inner part of the nucleus, consistent with previously described observations [28,32,38]. We believe that our results will open new grounds for research in the field of cancer genome organization, sheding some light into the mechanisms leading to gene repositioning and gene regulation in tumor initiation and progression. 

## Figures and Tables

**Table 1:** Clinical and cytogenetic features of the patients analyzed in this study.

Pt	Diagnosis	Karyotype	Reference
1	CMML	46,del(7)(q21q36)	[61]
2	MDS	46,XY,del(7)(q21.3-22q36)	[58,61]
3	AML-M5	46,XX,del(7)(q22q36)	[58]
4	MDS	46,XY,del(7)(q22q36)	[61]
5	MDS	45,XX,del(5)(q14),del(7)(q22), del(9)(q22),-17	This study
6	CML	47,XY,del(7)(q21),+21[20] ^1^	This study
7	FA	46,XY,dup(1)(q24q44),del(7)(q21)[5] ^1^	This study
8	AML-M6	44,XY,t(1;3)(p21;?),-5,del(7)(q31.1),-17,-20,+mar[cp18] ^1^	This study
9	t-AML	46,XX,del(7)(q32.1[4]/46,XX[6]	This study
10	AML	47,XY,+8[11]/47,idem,del(7)(q32.1)[9]	This study

Notes: Pt: Patient; CMML: Chronic myelomonocytic leukemia, MDS: Myelodysplastic syndrome; AML-M5: Acute myeloid leukemia M5 subtype, CML: Chronic myeloid leukemia, FA: Fanconi anemia, AML-M6: Acute myeloid leukemia M6 subtype, while also t-AML represents therapy-related acute myeloid leukemia.Archival samples from these patients were originally contributed by the Oncogenetic Laboratory, Children’s Hospital, University of Giessen, Germany (Pt. 1 to 4) and by the Pediatric Hematology Department, San Gerardo Hospital, Monza, Italy (Pt-5). ^1^ Original karyotypes were refined byfluorescence in situ hybridization (FISH) (see Table 2).

**Table 2:** Fluorescence in situ hybridization (FISH) mapping of chromosome 7 deletion breakpoints.

Probes	Band	Patients									
		Pt.1	Pt.2	Pt.3	Pt.4	Pt.5	Pt.6	Pt.7	Pt.8	Pt.9	Pt.10
RP11-90N9	7q21.11						+	+	+	+	+
Cos 5.2	7q21.3		+								
RP11-213E22	7q22.1						−	−	+	+	+
Cos 7d.2	7q22.1		−								
Cos 8a.2	7q22.1	−									
Cos 1120	7q22.1	−									
PAC 1065 (*CUTL1*)	7q22.1			+							
*KMT2E* gene ^1^	7q22						−	−	+	+	+
HSC7E485	7q22.2		−	−							
*MET* gene ^1^	7q31.2						−	−	−	+	+
RP11-73H23	7q34						−	−	−	−	−
HSC7E124	7q36.1		−	−							
*EZH2* gene ^1^	7q36.1						+	−	−	+	+
HSC7E86	7q36.2		−	+							
HSC7E769	7q36.2		+								
RP5-1121A15	7q36.3	+	+	+	+	+	+	+	+	+	+

Notes: The probes listed in this table have been described before [10,58]. + and − indicate retention or loss of the relative genomic region on the deleted chromosome 7. ^1^ probes from MetaSystems; empty spaces in correspondence to a probe, indicate no data available regarding retention or loss of the locus.

**Table 3:** *HLXB9* gene positioning and expression in the patients analyzed in this study.

*HLXB9*Expression	RNL	GC-level ^(a)^	Pt ID	
Not Expressed	0.754^(^•^)^	GC-poor	7	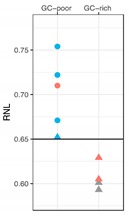
Not Expressed	0.722^(^•^)^	GC-poor	9	
Expressed	0.710^(^•^)^	GC-poor	10	
Not Expressed	0.671^(^•^)^	GC-poor	6	
NA	0.652^(▲)^	GC-poor	1	
Not Expressed	0.652^(▲)^	GC-poor	2	
Expressed	0.629^(▲)^	GC-rich	4	
Expressed	0.605^(▲)^	GC-rich	8	
NA	0.601^(▲)^	GC-rich	3	
NA	0.593^(▲)^	GC-rich	5	

Notes: Pt: Patient; RNL: Radial nuclear location evaluated by the median values of the nuclear location obtained in a large number of cells (see Material and Methods); *HLXB9* expr.: Expression evaluated by reverse transcription polymerase chain reaction (RT-PCR); NA: Data not available. (**a**) GC-level of the chromosomal region proximal to the centromeric breakpoint. (**right graph**) (^▲^) RNL of the two *HLXB9* alleles. (•) RNL of the *HLXB9* allele in the deleted chromosome 7. Plot color codes for gene expression: Blue - not expressed, red – expressed, gray – no information available (NA).

## Supplementary Materials

The following are available online at https://www.mdpi.com/2072-6694/11/4/585/s1, Figure S1: Distribution of *HLXB9* expression levels across datasets stratified by gender, Figure S2: *HLXB9* expression levels in AML, ALL, Whole Blood and Leukemia Cell Line differentiated per dataset of origin: GTEX, TARGET and TCGA, Figure S3: Distribution of *HLXB9* expression levels in TCGA AML data stratified per cytogenetic phenotype, Table S1: Phenotypic Characterization for del(7q) samples with high *HLXB9* expression levels.
