# Supplementary material for: Deletions of Chromosome 7q Affect Nuclear Organization and HLXB9Gene Expression in Hematological Disorders
Source: Cancers (Basel). 2019 Apr 25;11(4):585. doi: 10.3390/cancers11040585 (PMC6521283; doi:10.3390/cancers11040585)
Supplement: Supplementary file 1 [file cancers-11-00585-s001.pdf]

**Supplementary figures:**

**Table S1. Phenotypic characterisation for del(7q) samples with high *HLXB9* expression levels.**

<table>
<tr><th>Sample</th><th>HLXB9</th><th>Primary site</th><th>Gender</th><th>FISH test component</th><th>FISH test component percentage value</th><th>OS.time</th><th>OS</th><th>OS.unit</th><th>AML cytogenetics risk category</th><th>Age at diagnosis</th><th>Cytogenetic abnormality</th><th>Other cytogenetic abnormality</th><th>Molecular Analysis Abnormality Testing Result</th></tr>
<tr><td>TCGA-AB-2847-03</td><td>12.0594</td><td>WBC</td><td>Male</td><td>CBF-Beta|-7 or del(7q)</td><td>0|63</td><td>608</td><td>1</td><td>days</td><td>Intermediate/ Normal</td><td>53</td><td>Normal|del (7q) / 7q-</td><td>NA</td><td>FLT3 Mutation Negative|IDH1 R132 Negative|IDH1 R140 Negative|IDH1 R172 Negative|Activating RAS Negative|NPMc Negative</td></tr>
<tr><td>TCGA-AB-2860-03</td><td>1.1759</td><td>WBC</td><td>Female</td><td>NA</td><td>NA</td><td>427</td><td>1</td><td>days</td><td>Poor</td><td>60</td><td>Complex (greater than or equal to 3 distinct abnormalities)|del (5q) / 5q-|del (7q) / 7q-</td><td>t(4;11)</td><td>FLT3 Mutation Negative|IDH1 R132 Negative|IDH1 R140 Negative|IDH1 R172 Negative|Activating RAS Negative|NPMc Negative</td></tr>
<tr><td>TCGA-AB-2894-03</td><td>1.4395</td><td>WBC</td><td>Female</td><td>BCR-ABL|PML-RAR|MLL|CBF-Beta|TEL-AML 1|+8|-7 or del(7q)</td><td>0|0|74|0|0|0|76</td><td>181</td><td>1</td><td>days</td><td>Poor</td><td>50</td><td>Normal|del (7q) / 7q-|t (9;11)</td><td>NA</td><td>FLT3 Mutation Negative|IDH1 R132 Negative|IDH1 R140 Negative|IDH1 R172 Negative|Activating RAS Negative|NPMc Negative</td></tr>
<tr><td>TCGA-AB-2917-03</td><td>2.697</td><td>WBC</td><td>Female</td><td>NA</td><td>NA</td><td>1216</td><td>0</td><td>days</td><td>Poor</td><td>41</td><td>Normal|Complex (greater than or equal to 3 distinct abnormalities)|del (7q) / 7q-</td><td>NA</td><td>FLT3 Mutation Negative|IDH1 R132 Negative|IDH1 R140 Negative|IDH1 R172 Negative|Activating RAS Positive|NPMc Negative</td></tr>
<tr><td>TCGA-AB-2935-03</td><td>2.0142</td><td>WBC</td><td>Male</td><td>-7 or del(7q)</td><td>45</td><td>61</td><td>1</td><td>days</td><td>Poor</td><td>66</td><td>del (5q) / 5q-|del (7q) / 7q-</td><td>NA</td><td>BCR-ABL Negative|FLT3 Mutation Negative|IDH1 R132 Negative|IDH1 R140 Negative|IDH1 R172 Negative|Activating RAS Negative|NPMc Negative</td></tr>
<tr><td>TCGA-AB-2952-03</td><td>1.8336</td><td>WBC</td><td>Female</td><td>BCR-ABL|PML-RAR|+8</td><td>0|0|38</td><td>31</td><td>1</td><td>days</td><td>Poor</td><td>60</td><td>Complex (greater than or equal to 3 distinct abnormalities)|del (5q) / 5q-|del (7q) / 7q-|Trisomy 8</td><td>NA</td><td>FLT3 Mutation Negative|IDH1 R132 Negative|IDH1 R140 Negative|IDH1 R172 Negative|Activating RAS Negative|NPMc Negative</td></tr>
<tr><td>TCGA-AB-2987-03</td><td>1.4362</td><td>WBC</td><td>Female</td><td>BCR-ABL|PML-RAR|MLL|CBF-Beta|AML1-ETO|+8|-7 or del(7q)</td><td>0|0|0|0|0|0|0</td><td>184</td><td>1</td><td>days</td><td>Intermediate/ Normal</td><td>75</td><td>Normal|del (7q) / 7q-</td><td>NA</td><td>FLT3 Mutation Negative|IDH1 R132 Negative|IDH1 R140 Negative|IDH1 R172 Negative|Activating RAS Positive|NPMc Positive</td></tr>
</table>

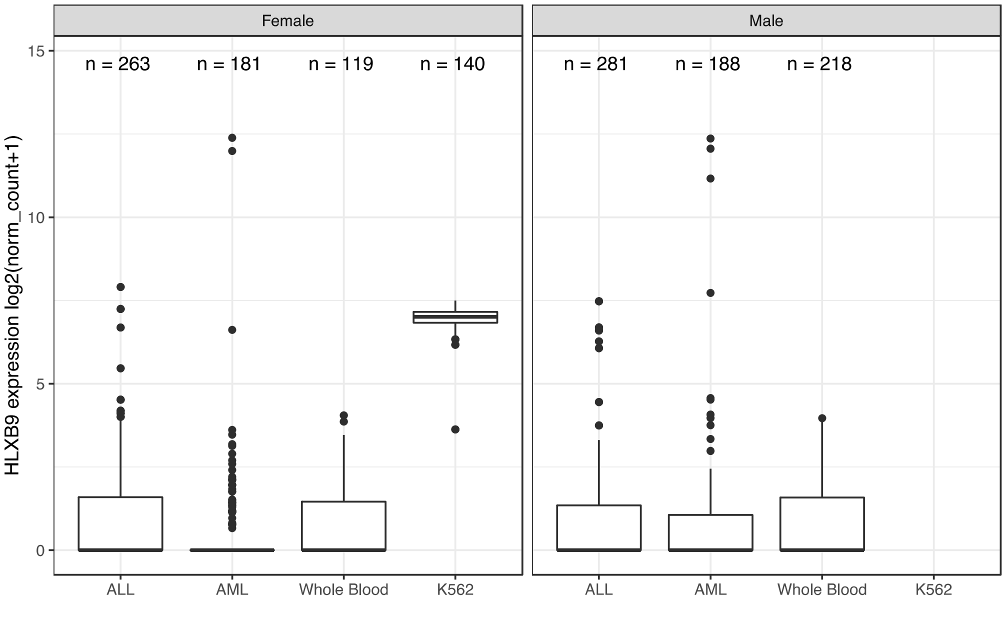


Figure S1. Distribution of *HLXB9* expression levels across datasets stratified by gender. "n" represents the number of samples in each category. The relationship between *HLXB9* expression and gender was analysed for all cohorts. We observed that there was no significant difference in the *HLXB9* expression levels between male and female samples.

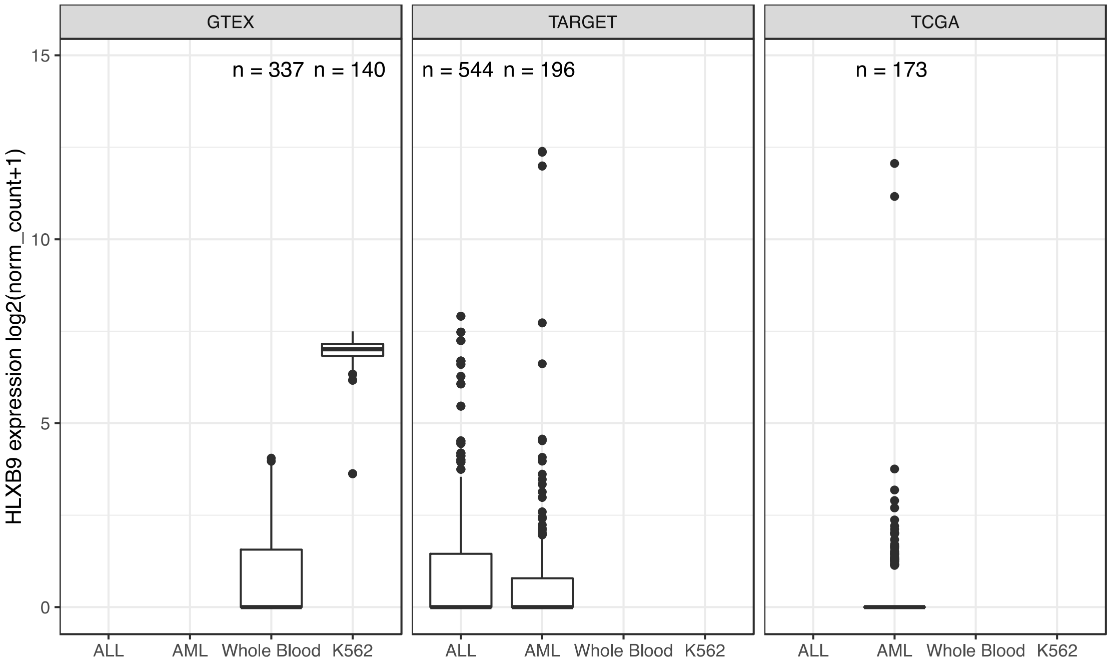


Figure S2. *HLXB9* expression levels in AML, ALL, Whole Blood and Leukemia Cell Line differentiated per dataset of origin: GTEX, TARGET and TCGA.

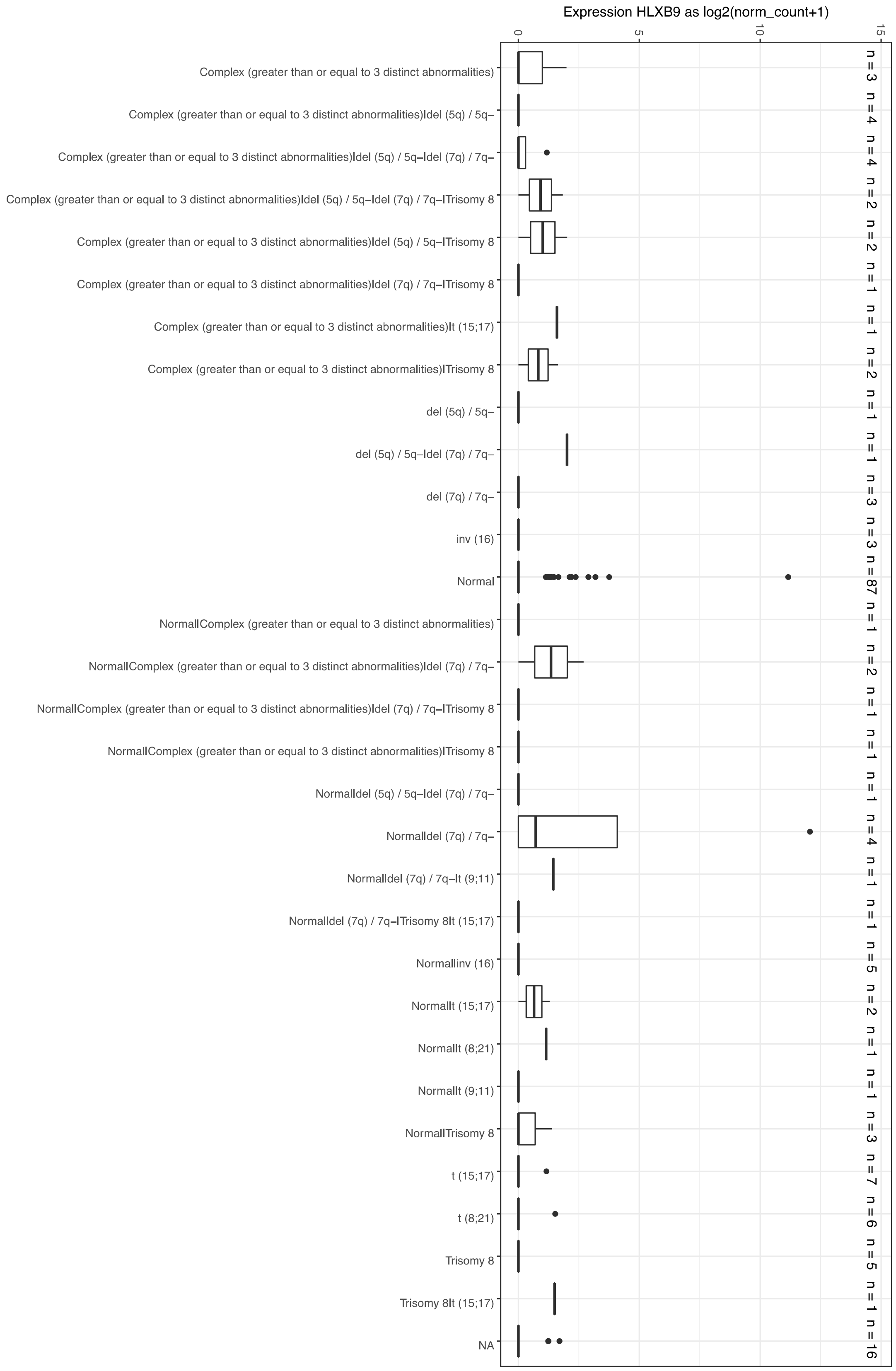


***Figure S3. Distribution of* HLXB9 *expression levels in TCGA AML data stratified per cyogenetic phenotype. "n" signifies the number of samples in each class.***
